# Long-Range Dependence in Word Time Series: The Cosine Correlation of Embeddings

**DOI:** 10.3390/e27060613

**Published:** 2025-06-09

**Authors:** Paweł Wieczyński, Łukasz Dębowski

**Affiliations:** 1 Independent Researcher, 80-180 Gdańsk, Poland; wieczynskipawel@gmail.com; 2 Institute of Computer Science, Polish Academy of Sciences, 01-248 Warsaw, Poland

**Keywords:** word embeddings, mutual information, cosine similarity, power laws, stretched exponential, long-range dependence

## Abstract

We analyze long-range dependence (LRD) for word time series, understood as a slower than exponential decay of the two-point Shannon mutual information. We achieve this by examining the decay of the cosine correlation, a proxy object defined in terms of the cosine similarity between word2vec embeddings of two words, computed by an analogy to the Pearson correlation. By the Pinsker inequality, the squared cosine correlation between two random vectors lower bounds the mutual information between them. Using the Standardized Project Gutenberg Corpus, we find that the cosine correlation between word2vec embeddings exhibits a readily visible stretched exponential decay for lags roughly up to 1000 words, thus corroborating the presence of LRD. By contrast, for the Human vs. LLM Text Corpus entailing texts generated by large language models, there is no systematic signal of LRD. Our findings may support the need for novel memory-rich architectures in large language models that exceed not only hidden Markov models but also Transformers.

## 1. Introduction

Consider a time series ${\left(W_{i}\right)}_{i\in Z}$ such as a text in natural language, a sequence of real numbers, or a sequence of vectors. Let $I(W_{i};W_{i+n})$ be the Shannon mutual information between two random variables separated by *n* positions. By short-range dependence (SRD), we understand an asymptotic exponential bound for the decay of this dependence measure,

(1) $$\begin{array}{c} I\left(W_{i};W_{i+n}\right)=O\left(\exp\left(-\delta n\right)\right),\;\delta >0. \end{array}$$

By long-range dependence (LRD), we understand any sort of decay of the dependence measure that does not fall under (1). In particular, under LRD, we may have a power-law decay of the dependence measure,

(2) $$\begin{array}{c} I\left(W_{i};W_{i+n}\right)∼n^{-\gamma },\;\gamma >0, \end{array}$$

which resembles a more standard definition of LRD for the autocorrelation function by Beran [1], or we may have a stretched exponential decay thereof,

(3) $$\begin{array}{c} I\left(W_{i};W_{i+n}\right)∼\exp\left(-\delta n^{\beta }\right),\;\delta >0,\;0<\beta <1. \end{array}$$

The SRD is characteristic of mixing Markov and hidden Markov processes ([2], Theorem 1), which assume that the probability of the next token depends only on a finite number of preceding tokens or on a bounded memory. Hence, the observation of LRD for sufficiently large lags implies that the time series generation cannot be modeled by a mixing Markov process of a relatively small order or—via the data-processing inequality ([3], Chapter 2.8)—by a mixing hidden Markov process with a small number of hidden states.

By contrast, it has often been expressed that texts in natural language exhibit LRD [2,4,5,6,7,8,9,10,11]. Several empirical studies analyzing textual data at different linguistic levels, such as characters [2,4], words [9], or punctuation [11], have indicated that correlations in natural language persist over long distances. This persistent correlation suggests that dependencies in human language extend far beyond adjacent words or short phrases, spanning across entire paragraphs or even longer discourse structures.

The LRD should be put on par with other statistical effects signaling that natural language is not a finite-state hidden Markov process, a theoretical linguistic claim that dates back to [12,13,14]. Let us write blocks of words $W_{j}^{k}:=\left(W_{j},W_{j+1},...,W_{k}\right)$. A power-law growth of the block mutual information

(4) $$\begin{array}{c} I\left(W_{1}^{n};W_{n+1}^{2n}\right)∼n^{\beta },\;0<\beta <1, \end{array}$$

is known as Hilberg’s law or as the neural scaling law [15,16,17]. Another observation [18] is a power-law logarithmic law of the maximal repetition length

(5) $$\begin{array}{c} L\left(W_{1}^{n}\right)∼{\left(\log n\right)}^{\alpha },\;\alpha >1, \end{array}$$

where we denote the maximal repetition length

(6) $$\begin{array}{c} L\left(W_{1}^{n}\right):=\max\left\{k\geq 1:W_{i+1}^{i+k}=W_{j+1}^{j+k}\;\mathrm{for}\;\mathrm{some}\;0\leq j<i\leq n-k\right\}. \end{array}$$

The long-range dependence (2) or (3), Hilberg’s law (4), and the maximal repetition law (5) have been all reported for natural language, whereas it can be mathematically proved that none of them is satisfied by finite-state hidden Markov processes [19,20].

The LRD, Hilberg’s law, and the maximal repetition law independently—and for different reasons—support the necessity of using complex memory architectures in contemporary large language models (LLMs). Neural networks designed for natural language processing must incorporate mechanisms capable of mimicking these laws. The older generation *n*-gram models struggle with this requirement for reasons that can be analyzed mathematically. By contrast, it is has been unclear whether Transformers [21], with their attention-based mechanisms, can leverage these extensive relationships. Understanding the nature of the LRD, Hilberg’s law, and the maximal repetition law in textual data may shed some light onto neural architectures that can progress on language modeling tasks.

Various smoothing techniques were proposed to discern LRD at the character or phoneme level [2,4,6,7]. Under no advanced estimation, the power-law decay of the Shannon mutual information between two characters dissolves into noise for lags up to 10 characters [4]. By contrast, Lin and Tegmark [2] considered sophisticated estimation techniques and reported the power-law decay of the Shannon mutual information between two characters for much larger lags.

Because of the arbitrariness of word forms relative to the semantic content of the text, we are not convinced that the results by Lin and Tegmark [2] are not an artifact of their estimation method. For this reason, following the idea of Mikhaylovskiy and Churilov [9], we have decided to seek the LRD on the level of words. We have supposed that pairs of words rather than pairs of characters better capture the long-range semantic coherence of the text. For this reason, we have expected that the LRD effect extends for a larger distance on the level of words than on the level of characters. Indeed, in the present study, we report a lower bound on the Shannon mutual information between two words that is salient for lags up to 1000 words, which is four decades of magnitude larger than the unsmoothed effect for characters.

A modest goal of this paper is to systematically explore a simple measure of dependence to check whether texts in natural language and those generated by large language models exhibit the LRD. Rather than directly investigating the Shannon mutual information, which is difficult to estimate for large alphabets and strongly dependent sources, we elect a measure of dependence called the cosine correlation. This object is related to the cosine similarity of two vectors and somewhat resembles the Pearson correlation. Formally, the cosine correlation between two random vectors *U* and *V* equals

(7) $$\begin{array}{c} \mathrm{CC}\left(U;V\right):=E\frac{U}{∥U∥}\cdot \frac{V}{∥V∥}-E\frac{U}{∥U∥}\cdot E\frac{V}{∥V∥}, \end{array}$$

where EX is the expectation of random variable *X*, U·V is the dot product, and $∥U∥:=\sqrt{U\cdot U}$ is the norm. By contrast, the cosine similarity of two non-random vectors *u* and *v* is

(8) $$\begin{array}{c} \cos\left(u;v\right):=\frac{u}{∥u∥}\cdot \frac{v}{∥v∥}. \end{array}$$

In order to compute the cosine correlation or the cosine similarity for actual word time series, we need a certain vector representation of words. As a practical vector representation of words, one may consider word2vec embeddings used in large language models [22,23]. Word embeddings capture semantic relationships between words by mapping them into continuous spaces, allowing for a more meaningful measure of similarity between distant words in a text. In particular, Mikhaylovskiy and Churilov [9] observed an approximate power-law decay for the expected cosine similarity Ecos(U;V) of word embeddings.

The paper by Mikhaylovskiy and Churilov [9] lacked, however, the following important theoretical insight. As a novel result of this paper, we demonstrate that the cosine correlation CC(U;V), rather than the expected cosine similarity Ecos(U;V), provides a lower bound for the Shannon mutual information I(U;V). Applying the Pinsker inequality [24,25], we obtain the bound

(9) $$\begin{array}{cc} I(U;V) & \geq \frac{\mathrm{CC}{\left(U;V\right)}^{2}}{2}. \end{array}$$

This approach provides an efficient alternative to direct statistical estimation of mutual information, which is often impractical due to the sparse nature of natural language data. In particular, a slower than exponential decay of the cosine correlation implies LRD. Thus, a time series with a power-law or stretched exponential decay of the cosine correlation is not a Markov process or a hidden Markov process.

Indeed, on the experimental side, we observe a stretched exponential decay of the cosine correlation, which is clearly visible roughly for lags up to 1000 words—but only for natural texts. By contrast, artificial texts do not exhibit this trend in a systematic way. Our source of natural texts is the Standardized Project Gutenberg Corpus [26], a diverse collection of literary texts that offers a representative sample of human language usage. Our source of artificial texts is the Human vs. LLM Text Corpus [27]. To investigate the effect of semantic correlations, we also consider the cosine correlation between moving sums of neighboring embeddings, a technique that we call pooling. Curiously, pooling does not make the stretched exponential decay substantially slower. The lack of a prominent LRD signal was already noticed for the previous generation of language models by Takahashi and Tanaka-Ishii [6,7].

Our observation of the slow decay of the cosine correlation in general confirms the prior results of Mikhaylovskiy and Churilov [9] and supports the hypothesis of LRD. We notice that Mikhaylovskiy and Churilov [9] did not try to fit the stretched exponential decay to their data and their power-law model was not visually very good. Both theoretical and experimental findings of this paper contribute to the growing body of statistical evidence proving that natural language is not a finite-state hidden Markov process.

What is more novel is that our findings may support the view that natural language cannot be either generated by Transformer-based large language models—in view of no systematic decay trend of the cosine correlation for the Human vs. LLM Text Corpus. As mentioned, the LRD, Hilberg’s law, and the maximal repetition law independently substantiate the necessity of sophisticated memory architectures in modern computational linguistic applications. These results open avenues for further research into the theoretical underpinnings of language structure, potentially informing the development of more effective models for language understanding and generation.

The organization of the article is as follows. Section 2 presents the theoretical results. Section 3 discusses the experiment. In particular, Section 3.1 presents our data. Section 3.2 describes the experimental methods. Section 3.3 presents the results. Section 3.4 offers the discussion. Section 4 contains the conclusion.

## 2. Theory

Similarly as Mikhaylovskiy and Churilov [9] but differently than Li [4], Lin and Tegmark [2], and Takahashi and Tanaka-Ishii [6,7], we will seek for LRD on the level of words rather than on the level of characters or phonemes. The Shannon mutual information between words is difficult to estimate for large alphabets and strongly dependent sources. Thus, we consider its lower bound defined via the cosine correlation of word2vec embeddings [22,23].

Let EX:=∫XdP denote the expectation of a real random variable *X*. Let lnx be the natural logarithm of *x* and let $H\left(X\right):=E\left[-\ln p(X)\right]$ be the Shannon entropy of a discrete random variable *X*, where p(X) is the probability density of *X* with respect to a reference measure ([3], Chapters 2.1 and 8.1). The Shannon mutual information between variables *X* and *Y* ([3], Chapters 2.4 and 8.5) equals

(10) $$\begin{array}{c} I(X;Y):=H(X)+H(Y)-H(X,Y) \end{array}$$

By contrast, the Pearson correlation between real random variables *X* and *Y* is defined as

(11) $$\begin{array}{c} \mathrm{Corr}\left(X;Y\right):=\frac{\mathrm{Cov}(X;Y)}{\sqrt{\mathrm{Var}(X)\mathrm{Var}(Y)}}. \end{array}$$

where we denote the covariance Cov(X;Y):=EXY−EXEY and the variance Var(X):=Cov(X;X). By the Cauchy–Schwarz inequality, we have |Corr(X;Y)|≤1.

We will introduce an analog of the Pearson correlation coefficient for vectors, which we call the cosine correlation. First, let us recall three standard concepts. For vectors $u=(u_{1},u_{2},...,u_{d})$ and $v=(v_{1},v_{2},...,v_{d})$, we consider the dot product

(12) $$\begin{array}{c} u\cdot v:=\sum_{k=1}^{d}u_{k}v_{k}, \end{array}$$

the norm $∥u∥:=\sqrt{u\cdot u}$, and the cosine similarity

(13) $$\begin{array}{c} \cos\left(u;v\right):=\frac{u}{∥u∥}\cdot \frac{v}{∥v∥}. \end{array}$$

By the Cauchy–Schwarz inequality, we have |cos(u;v)|≤1.

Now, we consider something less standard. For vector random variables *U* and *V*, we define the cosine correlation

(14) $$\begin{array}{cc} \mathrm{CC}(U;V):\; & =E\frac{U}{∥U∥}\cdot \frac{V}{∥V∥}-E\frac{U}{∥U∥}\cdot E\frac{V}{∥V∥}\\ \; & =E\left[\frac{U}{∥U∥}-E\frac{U}{∥U∥}\right]\cdot \left[\frac{V}{∥V∥}-E\frac{V}{∥V∥}\right]. \end{array}$$

If *U* and *V* are discrete and we denote the difference of measures

(15) $$\begin{array}{c} \Delta (u,v):=P(U=u,V=v)-P(U=u)P(V=v) \end{array}$$

then we may write

(16) $$\begin{array}{c} \mathrm{CC}\left(U;V\right)=\sum_{u,v}\Delta \left(u,v\right)\cos\left(u;v\right). \end{array}$$

We observe that if random variables *U* and *V* are unidimensional, then cos(U,V)=1 with probability 1 and CC(U;V)=0. Similarly, CC(U;V)=0 if cos(U,V) is constant with probability 1 or if *U* and *V* are independent.

To build some more intuitions, let us notice the following three facts. First of all, the cosine correlation between two copies of a random vector lies in the unit interval.

**Theorem** **1.** *We have*

(17) $$\begin{array}{c} 0\leq \mathrm{CC}(U;U)\leq 1. \end{array}$$

**Proof.**  Let us write $U^{\prime }:=U/∥U∥$. We have

(18) $$\begin{array}{cc} \mathrm{CC}(U;U)\; & =EU^{\prime }\cdot U^{\prime }-EU^{\prime }\cdot EU^{\prime }=1-\sum_{k=1}^{d}{\left(EU_{k}^{\prime }\right)}^{2}\\ \; & \geq 1-\sum_{k=1}^{d}E{\left(U_{k}^{\prime }\right)}^{2}=1-E\sum_{k=1}^{d}{\left(U_{k}^{\prime }\right)}^{2}=1-EU^{\prime }\cdot U^{\prime }=0. \end{array}$$

Hence, the claim follows.    □

Second, the cosine correlation satisfies a version of the Cauchy–Schwarz inequality.

**Theorem** **2.** *We have*

(19) $$\begin{array}{c} \left|\mathrm{CC}(U;V)\right|\leq \sqrt{\mathrm{CC}(U;U)\mathrm{CC}(V;V)}\leq 1. \end{array}$$

**Proof.**  Let us write $U^{\prime }:=U/∥U∥$ and $V^{\prime }:=V/∥V∥$. By the Cauchy–Schwarz inequalities $\left|\mathrm{Cov}(X;Y)\right|\leq \sqrt{\mathrm{Var}(X)\mathrm{Var}(Y)}$ for random scalars *X* and *Y* and $\sum_{k=1}^{d}\sqrt{x_{k}y_{k}}\leq \sqrt{\sum_{k=1}^{d}x_{k}}\sqrt{\sum_{k=1}^{d}y_{k}}$ for real numbers $x_{k},y_{k}\geq 0$, we obtain

(20) $$\begin{array}{cc} \left|\mathrm{CC}(U;V)\right| & \leq \sum_{k=1}^{d}\left|\mathrm{Cov}(U_{k}^{\prime };V_{k}^{\prime })\right|\leq \sum_{k=1}^{d}\sqrt{\mathrm{Var}\left(U_{k}^{\prime }\right)\mathrm{Var}\left(V_{k}^{\prime }\right)}\\ \; & \leq \sqrt{\sum_{k=1}^{d}\mathrm{Var}\left(U_{k}^{\prime }\right)}\sqrt{\sum_{k=1}^{d}\mathrm{Var}\left(V_{k}^{\prime }\right)}=\sqrt{\mathrm{CC}(U;U)\mathrm{CC}(V;V)}. \end{array}$$

Hence, the claim follows (17).    □

Third, we will show that cosine correlation CC(U;V) provides a lower bound for mutual information I(U;V).

**Theorem** **3.** *We have*

(21) $$\begin{array}{cc} I(U;V)\; & \geq \frac{\mathrm{CC}{\left(U;V\right)}^{2}}{2}. \end{array}$$

**Proof.**  Let us recall the Pinsker inequality

(22) $$\begin{array}{c} \sum_{x}p\left(x\right)\ln\frac{p(x)}{q(x)}\geq \frac{1}{2}{\left(\sum_{x}\left|p(x)-q(x)\right|\right)}^{2} \end{array}$$

for two discrete probability distributions *p* and *q* [24,25]. By the Pinsker inequality (22), the Cauchy–Schwarz inequality |cos(u;v)|≤1, and identity (16), we obtain

(23) $$\begin{array}{cc} I(U;V)\; & \geq \frac{1}{2}{\left(\sum_{u,v}\left|\Delta (u,v)\right|\right)}^{2}\geq \frac{1}{2}{\left(\sum_{u,v}\left|\Delta (u,v)||\cos(u,v)\right|\right)}^{2}\\ \; & \geq \frac{1}{2}{\left(\sum_{u,v}\Delta \left(u,v\right)\cos\left(u,v\right)\right)}^{2}=\frac{\mathrm{CC}{\left(U;V\right)}^{2}}{2}. \end{array}$$

Hence, the claim follows.    □

We note in passing that the Pinsker inequality can be modified as the Bretagnolle–Huber bound

(24) $$\begin{array}{c} \sum_{x}p\left(x\right)\ln\frac{p(x)}{q(x)}\geq -\ln\left(1-\frac{1}{4}{\left(\sum_{x}\left|p(x)-q(x)\right|\right)}^{2}\right) \end{array}$$

for probability distributions *p* and *q* [28,29]. Respectively, we obtain

(25) $$\begin{array}{cc} I(U;V)\; & \geq -\ln\left(1-\frac{\mathrm{CC}{\left(U;V\right)}^{2}}{4}\right). \end{array}$$

This bound is weaker than (21) since |CC(U;V)|≤1.

Let ${\left(W_{i}\right)}_{i\in Z}$ be the text in natural language treated as a word time series. Let $\phi \left(w\right)=(\phi_{1}\left(w\right),\phi_{2}\left(w\right),\ldots ,\phi_{d}\left(w\right))$ be an arbitrary vector representation of word *w*, such as word2vec embeddings [22,23], and let $F_{i}:=\left(F_{i1},F_{i2},\ldots ,F_{id}\right):=\phi \left(W_{i}\right)$. In particular, since embeddings $F_{i}=\phi \left(W_{i}\right)$ are functions of words $W_{i}$, by the data-processing inequality ([3], Chapter 2.8) and by the cosine correlation bound (21), we obtain

(26) $$\begin{array}{cc} I\left(W_{i};W_{j}\right)\geq I\left(F_{i};F_{j}\right)\; & \geq \frac{\mathrm{CC}{\left(F_{i};F_{j}\right)}^{2}}{2}. \end{array}$$

Wrapping up, a slow decay of cosine correlation $\mathrm{CC}(F_{i};F_{i+n})$ implies a slow decay of mutual information $I(W_{i};W_{i+n})$. Since $I(W_{i};W_{i+n})$ is damped exponentially for any mixing Markov or hidden Markov process ${\left(W_{i}\right)}_{i\in Z}$ by Theorem 1 of Lin and Tegmark [2], observing a power-law or a stretched exponential decay of cosine correlation $\mathrm{CC}(F_{i};F_{i+n})$ is enough to demonstrate that process ${\left(W_{i}\right)}_{i\in Z}$ is not a mixing Markov or hidden Markov process.

The framework that we have constructed in this section has its prior in the literature. We remark that Mikhaylovskiy and Churilov [9] investigated estimates of expectation $E\cos(F_{i};F_{i+n})$ rather than cosine correlation $\mathrm{CC}(F_{i};F_{i+n})$. That approach required estimation and subtraction of the asymptotic constant term. Mikhaylovskiy and Churilov [9] observed an approximate power-law decay but they did not mention the cosine correlation bound (21) in their discussion explicitly.

## 3. Experiment

### 3.1. Data

Our data consisted of three elements: a dictionary of embedding vectors for a subset of human languages, a corpus of texts written by humans in these languages, and a corpus of texts in English created by artificial intelligence. The considered set of human languages included 17 languages. Originally, we planned to use 20 languages with the largest text counts in the considered corpora but three of them, Esperanto, Chinese, and Tagalog, had to be excluded because the embedding dictionary did not cover these languages.

In particular, the source of pretrained word embeddings was chosen as the NLPL repository [23]. To provide a uniform baseline across languages, for all considered languages, we used 100-dimensional embedding vectors trained on the CoNLL17 corpora with the same algorithm, being the word2vec continuous skipgram algorithm. None of these embedding vector spaces includes lemmatization. The vocabulary sizes of the embedding spaces for the considered 17 languages are presented in Table 1.

As the source of texts written by humans, we chose the Standardized Project Gutenberg Corpus (SPGC) [26]. The corpus provides texts after some preprocessing and tokenization, as detailed in [26]. We filtered the SPGC to obtain a more manageable yet representative subset of texts. As we have mentioned, we restricted the corpus to 17 languages with the largest text counts simultaneously covered by the applied NLPL embedding dictionary. Moreover, we filtered out files of the size above 1000 KB and we sampled up to 100 texts (or fewer if not available) per language in order to achieve roughly balanced subsets across particular languages.

To provide a comparison with texts generated by artificial intelligence, we also considered the Human vs. LLM Text Corpus (HLLMTC) [27]. All texts in the HLLMTC are in English. To make this corpus more easily computationally tractable, we sampled 1000 human written texts and 6000 LLM generated texts, where we chose 1000 texts per each of the six selected large language models. To convert these texts into word time series, we used off-the-shelf tokenizer [30].

Table 2 provides the summary statistics of the obtained subsets of the Standardized Project Gutenberg Corpus and the Human vs. LLM Text Corpus. In particular, we report the token counts and the coverage of the sampled texts, i.e., the fraction of word tokens of texts that appear in the respective NLPL embedding dictionary.

### 3.2. Methods

In this section, we briefly describe what we measured and in what way. We supposed that the LRD on the level of words is due to semantic coherence of the text over longer distances. In particular, mutual information between two words is large as long as the text around these words concerns a similar topic. We supposed that the embedding of this local topic can be roughly estimated as the sum of embeddings of all words in the neighborhood, called a pooled embedding. Let $F_{i}$ be the embedding of the *i*-th word in the text. The pooled embeddings are defined as

(27) $$\begin{array}{c} F_{i}^{\left(k\right)}:=\sum_{j=0}^{k-1}F_{i+j} \end{array}$$

for the pooling order k≥1. In particular, pooled embeddings for k=1 equal word embeddings, $F_{i}^{\left(1\right)}=F_{i}$.

The object that we wanted to measure was the cosine correlation for pooled embeddings, namely

(28) $$\begin{array}{c} C\left(n|k\right):=\mathrm{CC}\left(F_{i}^{\left(k\right)};F_{i+n}^{\left(k\right)}\right). \end{array}$$

Function C(n|k) is substantially larger for 0≤n<k since the summations for variables $F_{i}^{\left(k\right)}$ and $F_{i+n}^{\left(k\right)}$ range partly over overlapping embeddings $F_{i}$. Thus, if one wants to estimate the functional form of the decay of C(n|k), it makes sense to fit the respective function exclusively to data points where n≥k.

Let us proceed to the estimation of function C(n|k). Let ϕ(w) be the embedding of word *w* according to the considered word2vec dictionary. From each text, we removed all word tokens that did not have an embedding in the dictionary. In this way, we obtained a collection of word time series $\left(W_{1},W_{2},...,W_{N}\right)$, corresponding word embeddings $F_{i}=\phi \left(W_{i}\right)$, and pooled embeddings $F_{i}^{\left(k\right)}$ given by formula (27). We estimated the expectations as the averages over the times series. That is, we computed the estimator of C(n|k) defined as

(29) $$\begin{array}{c} \hat{C}\left(n|k\right):=\frac{1}{N-n}\sum_{i=1}^{N-n}U_{i}^{\left(k\right)}\cdot U_{i+n}^{\left(k\right)}, \end{array}$$

where we used the auxiliary time series

(30) $$\begin{array}{cc} U_{i}^{\left(k\right)} & :=\frac{F_{i}^{\left(k\right)}}{∥F_{i}^{\left(k\right)}∥}-\frac{1}{N}\sum_{j=1}^{N}\frac{F_{j}^{\left(k\right)}}{∥F_{j}^{\left(k\right)}∥}. \end{array}$$

We observe that $F_{i+1}^{\left(k\right)}=F_{i}^{\left(k\right)}-F_{i}+F_{i+k}$. Therefore, the computational complexity of estimator $\hat{C}\left(n|k\right)$ for fixed *n* and *k* is of order O(Nd), where *N* is the text length and *d* is the dimension of embeddings $F_{i}$.

For each text $\left(W_{1},W_{2},\ldots ,W_{N}\right)$, we computed estimators $\hat{C}\left(n|k\right)$ for lags n∈A∩[1,N], where

(31) $$\begin{array}{cc} A & :=\left\{1,\left\lceil 1.1\right\rceil ,\left\lceil 1.1^{2}\right\rceil ,\left\lceil 1.1^{3}\right\rceil ,\ldots \right\}, \end{array}$$

and pooling orders $k\in \left\{1,3,3^{2},3^{3}\right\}$. We observed that the plot of the absolute value $|\hat{C}\left(n|k\right)|$ for considered texts usually dissolved into random noise around n=1000 and there was a hump for n<k, as expected. Hence, to estimate the functional form of the decay of $|\hat{C}\left(n|k\right)|$, we restricted the fitting procedure to range k≤n≤1000.

The parameter estimation was performed using the curve_fit function from the SciPy library [31], which employs the trust region reflective algorithm. We selected this method due to its compatibility with bounded constraints. We estimated parameters of two functions: the power-law decay

(32) $$\begin{array}{cc} f(n|c,\gamma ) & :=cn^{-\gamma },\;c\in R,\;\gamma >0, \end{array}$$

and the stretched exponential decay

(33) $$\begin{array}{cc} f(n|b,\delta ,\beta ) & :=\exp(-\delta n^{\beta }+b),\;b\in R,\;\delta >0,\;0<\beta <1, \end{array}$$

with parameters γ, δ, and β implicitly depending on the pooling order *k*. As a goodness-of-fit metric, we calculated the sum of squared logarithmic residuals

(34) $$\begin{array}{cc} \mathrm{SSLR}:\; & =\sum_{n\in A\cap [k,1000]}{\left(\log|\hat{C}\left(n|k\right)|-\log f\left(n|\ldots \right)\right)}^{2} \end{array}$$

divided by the number of the degrees of freedom (ndf) equal to |A∩[k,1000]| minus the number of parameters of f(n|…).

We investigated the dependence of the results on the source, understood as the particular language for human-written texts or the particular language model for LLM-generated texts. To check whether there are significant differences of the distribution of a parameter $\alpha \in \left\{\gamma ,\delta ,\beta \right\}$ across particular sources, we used the non-parametric Kruskal–Wallis test with the null hypothesis

(35) $$\begin{array}{c} H_{0}:P_{1}=P_{2}=\ldots =P_{J}, \end{array}$$

where $P_{j}$ is the distribution of parameter α for the *j*-th source. To further explore differences among different sources, we employed the post-hoc Dunn test with the Bonferroni correction for multiple comparisons.

### 3.3. Results

**Figure 1:**
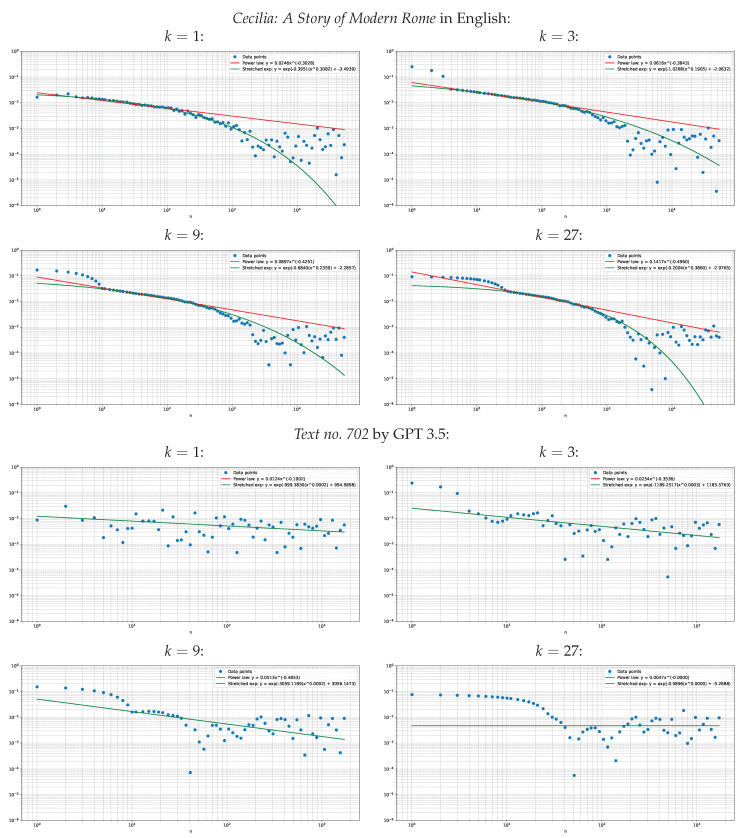
Estimates $|\hat{C}\left(n|k\right)|$ for two diagnostic texts.

Visually, the decay of the absolute cosine correlation estimates $|\hat{C}\left(n|k\right)|$ for k≤n≤1000 usually follows a stretched exponential form rather than the exact power-law decay for human-written text. By contrast, no systematic decay for k≤n can be detected for LLM-generated texts. This tendency can be seen in Figure 1, which is a diagnostic plot of the absolute cosine correlation estimates $|\hat{C}\left(n|k\right)|$ for two texts: *Cecilia: A Story of Modern Rome* in English from the SPGC corpus and *Text no. 702* by GPT 3.5, which is the longest LLM-generated text in the sampled subset of the HLLMTC corpus.

In Table 3, Table 4, Table 5, Table 6 and Table 7, we report the means and the standard deviations of the fitted parameters *c* and γ of the power-law model (32) and *b*, δ, and β of the stretched exponential model (33). The values are reported as they depend on a particular language for human-written texts or on a particular language model for LLM-generated texts. When fitting the models, the optimization algorithm did not converge sometimes. The failure rates and the overall goodness of fit are reported in Table 8. Despite the visual appeal of the stretched exponential model, the mean SSLR given by Formula (34) is less for the power-law model. This does not necessarily mean that the power-law model is better, however, since the standard deviation of the SSLR is greater than the mean for the stretched exponential model.

### 3.4. Discussion

Similarly as Mikhaylovskiy and Churilov [9] but differently than Li [4], Lin and Tegmark [2], and Takahashi and Tanaka-Ishii [6,7], we have sought for the LRD on the level of words rather than on the level of characters or phonemes. We have hypothesized that word-level dependencies yield a more prominent effect due to semantic coherence of lexical units over longer distances as compared to phoneme-level correlations, which tend to decay faster, in view of the arbitrariness of word forms.

Indeed, analyzing the cosine similarity of word embeddings, like Mikhaylovskiy and Churilov [9], or their cosine correlation, in the present study, one observes a clearly visible LRD effect for natural, i.e., human-written texts. Mikhaylovskiy and Churilov [9] reported a rough power-law decay without considering an alternative model. By contrast, we have considered both a power-law model and a stretched exponential model and both natural texts and LLM-generated texts.

We report that the slow decay of the cosine correlation extends up to 1000 words for natural texts, whereas it is dominated by noise for LLM-generated texts—as it was already observed for the previous generation of language models [6,7]. These effects can be seen in the diagnostic Figure 1 and independently witnessed by Table 3, Table 4, Table 5, Table 6, Table 7 and Table 8, where fitting to the random noise results in highly unstable estimates and outliers pumping up the standard deviations beyond the means. Curiously, the decay of the cosine correlation does not change systematically as the pooling order *k* increases, despite our prior expectation that the cosine correlation would increase monotonically with *k*.

The distributions of fitted parameters *c* and γ of the power-law model (32) and *b*, δ, and β of the stretched exponential model (33) vary significantly across different human languages and different large language models (p<0.01 for the Kruskall–Wallis tests). It means that the cosine correlation decays at different source-specific rates. At the moment, we are unable to state clearly what the cause for this variation may be.

For example, the Japanese language seems an outlier in many categories but this need not be directly caused by language typology. We notice that the available texts in Japanese are very short and their coverage in terms of embeddings is much lower than for other languages. Maybe our experimental methodology fails for very short texts in general. This might be an alternative explanation of the poor fitting results for LLM-generated texts which are very short, as well, as shown in Table 2 and Table 8.

## 4. Conclusions

In this paper, we have provided an empirical support for the claim that texts in natural language exhibit long-range dependence (LRD), understood as a slower than exponential decay of the two-point mutual information. Similar claims have been reiterated in the literature [2,4,5,6,7,8,9,10,11] but we hope that we have provided more direct and convincing evidence.

First, as a theoretical result, we have shown that the squared cosine correlation lower bounds the Shannon mutual information between two vectors. Under this bound, a power-law or a stretched exponential decay of the cosine correlation implies the LRD. In particular, the vector time series which exhibits such a slow decay of the cosine correlation cannot be not a mixing Markov or hidden Markov process by Theorem 1 of Lin and Tegmark [2].

Second, using the Standardized Project Gutenberg Corpus [26] and vector representations of words taken from the NLPL repository [23], we have shown experimentally that the estimates of the cosine correlation of word embeddings follow a stretched exponential decay. This decay extends for lags up to 1000 words without any smoothing, which is four decades of magnitude larger than the unsmoothed, presumably LRD, effect for characters [4].

Third, the stability of this decay suggests that the LRD is a fundamental property of natural language, rather than an artifact of specific preprocessing methods or statistical estimation techniques. The observation of the slow decay of the cosine correlation for natural texts not only supports the hypothesis of LRD but also reaffirms the prior results of Mikhaylovskiy and Churilov [9], who reported a rough power-law decay of the expected cosine similarity of word embeddings.

Fourth, like Takahashi and Tanaka-Ishii [6,7], we have observed the LRD only for natural data. We stress that, as we were able to observe, artificial data do not exhibit the LRD in a systematic fashion. Our source of artificial texts was the Human vs. LLM Text Corpus [27]. We admit that texts in this corpus may be too short to draw firm conclusions and further research on longer LLM-generated texts is necessary to confirm our early claim.

As we have mentioned in the introduction, non-Markovianity effects such as the LRD, Hilberg’s law [15,16,17], and the maximal repetition law [18] may have implications for understanding the limitations and capabilities of contemporary language models. The presence of such effects in natural texts in contrast to texts generated by language models highlights the indispensability of complex memory mechanisms, potentially showing that state-of-the-art architectures such as Transformers [21] are insufficient.

Future research might explore whether novel architectures could capture quantitative linguistic constraints such as the LRD more effectively [32]. Further studies may also explore alternative embeddings or dependence measures and their impact on the stability of the LRD measures such as the stretched exponential decay parameters. Investigating other linguistic corpora, text genres, and languages could also provide valuable insights into the universality of these findings.

## Figures and Tables

**Table 1:** Vocabulary sizes of the chosen embedding spaces.

Language	Vocabulary Size
Catalan (ca)	799,020
Danish (da)	1,655,886
German (de)	4,946,997
Greek (el)	1,183,194
English (en)	4,027,169
Spanish (es)	2,656,057
Finnish (fi)	2,433,286
French (fr)	2,567,698
Hungarian (hu)	2,702,663
Italian (it)	2,469,122
Japanese (ja)	3,989,605
Latin (la)	555,381
Dutch (nl)	2,610,658
Norwegian (no)	223,763
Polish (pl)	4,420,598
Portuguese (pt)	2,536,452
Swedish (sv)	3,010,472

**Table 2:** Summary of the used subset of corpora.

	# of Texts	# of Tokens		Coverage	
Source		Mean	Std	Mean	Std
Standardized Project Gutenberg Corpus:					
Catalan (ca)	32	36,827.44	20,376.28	0.97	0.02
Danish (da)	66	51,832.92	30,748.01	0.97	0.02
German (de)	100	41,532.84	32,192.86	0.97	0.02
Greek (el)	100	29,487.20	17,775.53	0.91	0.03
English (en)	100	47,362.22	41,189.09	1.00	0.01
Spanish (es)	100	62,873.16	37,040.17	0.98	0.02
Finnish (fi)	100	35,095.02	28,948.42	0.94	0.03
French (fr)	100	53,948.66	39,585.75	0.96	0.01
Hungarian (hu)	100	50,510.30	31,976.38	0.95	0.02
Italian (it)	100	54,386.01	37,917.55	0.95	0.03
Japanese (ja)	20	268.05	371.40	0.78	0.19
Latin (la)	76	26,769.57	31,199.93	0.93	0.07
Dutch (nl)	100	43,055.05	31,465.48	0.98	0.01
Norwegian (no)	19	39,497.00	24,798.87	0.93	0.03
Polish (pl)	29	14,225.28	16,859.88	0.96	0.06
Portuguese (pt)	100	18,485.80	18,533.72	0.96	0.01
Swedish (sv)	100	37,474.97	29,310.51	0.97	0.02
Human vs. LLM Text Corpus:					
GPT-3.5	1000	444.40	278.66	0.9996	0.0025
GPT-4	1000	628.69	228.94	0.9996	0.0023
Human	1000	666.63	881.15	0.9980	0.0055
LLaMA-13B	1000	437.87	268.76	0.9987	0.0058
LLaMA-30B	1000	404.65	261.39	0.9988	0.0053
LLaMA-65B	1000	369.19	252.73	0.9988	0.0061
LLaMA-7B	1000	489.27	263.58	0.9986	0.0070

**Table 3:** Means and standard deviations of parameter *c*.

Source	Pooling Order			
	k=1	k=3	k=9	k=27
Standardized Project Gutenberg Corpus:				
ca	0.0248 ± 0.0035	0.069 ± 0.013	0.099 ± 0.024	0.17 ± 0.12
da	0.0203 ± 0.0045	0.052 ± 0.013	0.080 ± 0.024	0.150 ± 0.091
de	0.0286 ± 0.0079	0.070 ± 0.021	0.108 ± 0.047	0.27 ± 0.67
el	0.031 ± 0.012	0.072 ± 0.024	0.100 ± 0.041	0.19 ± 0.20
en	0.0258 ± 0.0092	0.070 ± 0.026	0.108 ± 0.053	0.20 ± 0.18
es	0.033 ± 0.022	0.088 ± 0.037	0.120 ± 0.049	0.19 ± 0.12
fi	0.0501 ± 0.0082	0.100 ± 0.019	0.144 ± 0.046	0.24 ± 0.13
fr	0.033 ± 0.013	0.087 ± 0.033	0.127 ± 0.076	0.27 ± 0.57
hu	0.0353 ± 0.0058	0.086 ± 0.016	0.119 ± 0.030	0.21 ± 0.18
it	0.0327 ± 0.0093	0.085 ± 0.025	0.117 ± 0.047	0.19 ± 0.16
ja	0.148 ± 0.094	0.45 ± 0.39	3.5 ± 9.6	21,259 ± 72,964
la	0.067 ± 0.031	0.154 ± 0.076	0.24 ± 0.16	0.63 ± 0.96
nl	0.0257 ± 0.0078	0.071 ± 0.026	0.113 ± 0.057	0.24 ± 0.27
no	0.0136 ± 0.0051	0.040 ± 0.012	0.068 ± 0.019	0.110 ± 0.041
pl	0.046 ± 0.011	0.118 ± 0.025	0.179 ± 0.077	0.42 ± 0.48
pt	0.026 ± 0.011	0.087 ± 0.034	0.16 ± 0.12	0.32 ± 0.50
sv	0.027 ± 0.012	0.065 ± 0.022	0.101 ± 0.039	0.19 ± 0.14
Human vs. LLM Text Corpus:				
GPT-3.5	0.024 ± 0.022	0.05 ± 0.74	2 ± 57	5.4 $\times \;10^{30}$ ± 1.7 $\times \;10^{31}$
GPT-4	0.029 ± 0.019	0.1 ± 3.2	0.02 ± 0.52	−0.1 ± 1.2
Human	0.028 ± 0.021	0.039 ± 0.061	3 ± 59	1.0 $\times \;10^{7}$ ± 3.1 $\times \;10^{8}$
LLaMA-13B	0.027 ± 0.027	0.045 ± 0.088	2 ± 31	1.0 $\times \;10^{30}$ ± 3.1 $\times \;10^{31}$
LLaMA-30B	0.027 ± 0.024	0.040 ± 0.048	0.3 ± 4.8	2.6 $\times \;10^{19}$ ± 8.0 $\times \;10^{20}$
LLaMA-65B	0.026 ± 0.023	0.04 ± 0.18	1 ± 23	1.7 $\times \;10^{7}$ ± 4.4 $\times \;10^{8}$
LLaMA-7B	0.029 ± 0.027	0.044 ± 0.051	0.3 ± 4.0	6.2 $\times \;10^{11}$ ± 1.9 $\times \;10^{12}$

**Table 4:** Means and standard deviations of parameter γ.

Source	Pooling Order			
	k=1	k=3	k=9	k=27
Standardized Project Gutenberg Corpus:				
ca	0.449 ± 0.055	0.523 ± 0.063	0.546 ± 0.083	0.62 ± 0.13
da	0.373 ± 0.067	0.442 ± 0.092	0.49 ± 0.11	0.58 ± 0.17
de	0.440 ± 0.079	0.49 ± 0.11	0.53 ± 0.14	0.62 ± 0.23
el	0.405 ± 0.083	0.48 ± 0.13	0.51 ± 0.16	0.57 ± 0.23
en	0.330 ± 0.067	0.418 ± 0.098	0.47 ± 0.12	0.54 ± 0.19
es	0.373 ± 0.090	0.44 ± 0.11	0.45 ± 0.12	0.50 ± 0.16
fi	0.574 ± 0.084	0.552 ± 0.099	0.57 ± 0.13	0.62 ± 0.17
fr	0.415 ± 0.079	0.47 ± 0.11	0.49 ± 0.13	0.55 ± 0.20
hu	0.442 ± 0.075	0.482 ± 0.096	0.49 ± 0.11	0.55 ± 0.17
it	0.421 ± 0.089	0.47 ± 0.12	0.48 ± 0.13	0.52 ± 0.19
ja	0.34 ± 0.19	0.57 ± 0.39	0.79 ± 0.69	2.3 ± 1.6
la	0.40 ± 0.17	0.47 ± 0.23	0.51 ± 0.24	0.60 ± 0.33
nl	0.390 ± 0.074	0.46 ± 0.11	0.51 ± 0.13	0.60 ± 0.20
no	0.347 ± 0.041	0.451 ± 0.060	0.522 ± 0.073	0.59 ± 0.10
pl	0.550 ± 0.075	0.63 ± 0.12	0.65 ± 0.18	0.73 ± 0.26
pt	0.416 ± 0.071	0.57 ± 0.16	0.63 ± 0.22	0.70 ± 0.47
sv	0.407 ± 0.084	0.459 ± 0.095	0.51 ± 0.12	0.60 ± 0.17
Human vs. LLM Text Corpus:				
GPT-3.5	0.31 ± 0.36	0.19 ± 0.36	0.9 ± 1.9	1.2 ± 2.0
GPT-4	0.47 ± 0.30	0.39 ± 0.42	0.6 ± 1.3	0.8 ± 1.6
Human	0.28 ± 0.26	0.29 ± 0.38	0.7 ± 1.3	0.9 ± 1.3
LLaMA-13B	0.19 ± 0.20	0.25 ± 0.27	0.6 ± 1.3	0.8 ± 1.4
LLaMA-30B	0.20 ± 0.20	0.25 ± 0.35	0.6 ± 1.3	0.8 ± 1.3
LLaMA-65B	0.19 ± 0.20	0.23 ± 0.27	0.6 ± 1.2	0.8 ± 1.2
LLaMA-7B	0.21 ± 0.20	0.25 ± 0.24	0.6 ± 1.0	0.7 ± 1.1

**Table 5:** Means and standard deviations of parameter *b*.

Source	Pooling Order			
	k=1	k=3	k=9	k=27
Standardized Project Gutenberg Corpus:				
ca	0.9 ± 6.1	192 ± 524	26 ± 114	24 ± 127
da	119 ± 552	107 ± 490	69 ± 322	131 ± 571
de	137 ± 586	56 ± 246	134 ± 531	89 ± 480
el	126 ± 501	63 ± 206	128 ± 481	80 ± 335
en	93 ± 472	39 ± 157	108 ± 529	36 ± 183
es	147 ± 615	124 ± 334	34 ± 188	50 ± 255
fi	26 ± 40	151 ± 406	97 ± 419	127 ± 504
fr	81 ± 333	110 ± 478	121 ± 461	106 ± 466
hu	10 ± 28	40 ± 158	21 ± 130	53 ± 197
it	114 ± 483	86 ± 231	83 ± 402	108 ± 527
ja	195 ± 652	449 ± 992	252 ± 902	479 ± 1239
la	406 ± 1044	176 ± 672	231 ± 701	375 ± 1029
nl	4 ± 31	19 ± 101	14 ± 129	47 ± 257
no	271 ± 745	5 ± 27	−0.7 ± 3.9	84 ± 356
pl	157 ± 416	387 ± 1071	364 ± 725	610 ± 1189
pt	353 ± 915	338 ± 884	496 ± 1207	588 ± 1174
sv	114 ± 455	106 ± 534	28 ± 150	69 ± 455
Human vs. LLM Text Corpus:				
GPT-3.5	1575 ± 1629	709 ± 1156	421 ± 877	198 ± 590
GPT-4	2520 ± 1489	1749 ± 1510	1127 ± 1333	325 ± 793
Human	1211 ± 1462	724 ± 1144	624 ± 1126	581 ± 1149
LLaMA-13B	513 ± 951	502 ± 959	662 ± 1129	522 ± 1029
LLaMA-30B	569 ± 1002	469 ± 893	664 ± 1108	494 ± 993
LLaMA-65B	554 ± 982	479 ± 912	606 ± 1090	458 ± 987
LLaMA-7B	553 ± 1010	506 ± 946	719 ± 1173	526 ± 1062

**Table 6:** Means and standard deviations of parameter δ.

Source	Pooling Order			
	k=1	k=3	k=9	k=27
Standardized Project Gutenberg Corpus:				
ca	4.7 ± 6.0	195 ± 524	29 ± 114	27 ± 127
da	123 ± 552	110 ± 490	72 ± 322	134 ± 571
de	141 ± 586	59 ± 246	137 ± 531	92 ± 480
el	130 ± 501	66 ± 206	131 ± 481	83 ± 335
en	97 ± 472	42 ± 157	111 ± 529	39 ± 183
es	151 ± 615	127 ± 334	37 ± 188	52 ± 255
fi	29 ± 40	153 ± 406	100 ± 419	130 ± 504
fr	84 ± 333	112 ± 478	123 ± 461	108 ± 466
hu	13 ± 28	43 ± 158	23 ± 130	56 ± 197
it	118 ± 483	88 ± 231	86 ± 402	111 ± 527
ja	198 ± 652	451 ± 992	254 ± 900	480 ± 1238
la	409 ± 1044	178 ± 672	234 ± 701	377 ± 1028
nl	8 ± 31	21 ± 101	17 ± 129	50 ± 257
no	275 ± 744	8 ± 27	2.6 ± 3.8	87 ± 356
pl	160 ± 416	389 ± 1071	367 ± 725	612 ± 1188
pt	356 ± 915	341 ± 884	498 ± 1207	590 ± 1173
sv	118 ± 455	109 ± 534	31 ± 150	72 ± 455
Human vs. LLM Text Corpus:				
GPT-3.5	1579 ± 1628	714 ± 1155	426 ± 876	203 ± 589
GPT-4	2523 ± 1488	1753 ± 1509	1132 ± 1331	330 ± 792
Human	1214 ± 1462	728 ± 1144	627 ± 1125	584 ± 1148
LLaMA-13B	517 ± 951	506 ± 958	665 ± 1129	526 ± 1027
LLaMA-30B	573 ± 1001	473 ± 893	667 ± 1107	498 ± 992
LLaMA-65B	558 ± 982	483 ± 911	610 ± 1089	462 ± 986
LLaMA-7B	557 ± 1009	510 ± 946	723 ± 1172	530 ± 1060

**Table 7:** Means and standard deviations of parameter β.

Source	Pooling Order			
	k=1	k=3	k=9	k=27
Standardized Project Gutenberg Corpus:				
ca	0.16 ± 0.11	0.090 ± 0.093	0.16 ± 0.15	0.24 ± 0.21
da	0.23 ± 0.17	0.17 ± 0.11	0.21 ± 0.16	0.23 ± 0.19
de	0.14 ± 0.15	0.14 ± 0.12	0.18 ± 0.15	0.27 ± 0.19
el	0.18 ± 0.13	0.14 ± 0.15	0.18 ± 0.17	0.30 ± 0.24
en	0.28 ± 0.15	0.17 ± 0.14	0.21 ± 0.15	0.31 ± 0.23
es	0.17 ± 0.12	0.11 ± 0.15	0.15 ± 0.15	0.23 ± 0.20
fi	0.071 ± 0.067	0.067 ± 0.065	0.12 ± 0.11	0.21 ± 0.15
fr	0.14 ± 0.14	0.13 ± 0.15	0.16 ± 0.19	0.24 ± 0.23
hu	0.086 ± 0.069	0.093 ± 0.090	0.18 ± 0.16	0.26 ± 0.25
it	0.119 ± 0.095	0.10 ± 0.13	0.13 ± 0.15	0.20 ± 0.18
ja	0.61 ± 0.45	0.55 ± 0.49	0.59 ± 0.48	0.49 ± 0.48
la	0.20 ± 0.22	0.32 ± 0.25	0.40 ± 0.30	0.52 ± 0.37
nl	0.19 ± 0.15	0.15 ± 0.13	0.19 ± 0.15	0.27 ± 0.21
no	0.30 ± 0.18	0.22 ± 0.11	0.187 ± 0.081	0.23 ± 0.18
pl	0.070 ± 0.068	0.090 ± 0.089	0.15 ± 0.17	0.24 ± 0.24
pt	0.19 ± 0.16	0.13 ± 0.16	0.18 ± 0.20	0.23 ± 0.25
sv	0.16 ± 0.12	0.17 ± 0.13	0.21 ± 0.15	0.29 ± 0.24
Human vs. LLM Text Corpus:				
GPT-3.5	0.07 ± 0.24	0.11 ± 0.30	0.18 ± 0.37	0.28 ± 0.44
GPT-4	0.02 ± 0.12	0.04 ± 0.19	0.06 ± 0.22	0.11 ± 0.30
Human	0.10 ± 0.27	0.19 ± 0.35	0.27 ± 0.41	0.32 ± 0.44
LLaMA-13B	0.24 ± 0.38	0.26 ± 0.39	0.25 ± 0.40	0.32 ± 0.45
LLaMA-30B	0.21 ± 0.36	0.24 ± 0.38	0.24 ± 0.40	0.31 ± 0.44
LLaMA-65B	0.20 ± 0.36	0.23 ± 0.38	0.26 ± 0.42	0.33 ± 0.45
LLaMA-7B	0.25 ± 0.39	0.27 ± 0.39	0.27 ± 0.41	0.30 ± 0.44

**Table 8:** Failure rates and goodness of fit for power-law (PL) decay and stretched exponential (SE) decay.

Pooling Order	PL	PL	SE	SE
	Failure	Avg.	Failure	Avg.
	Rate (%)	SSLR	Rate (%)	SSLR
Standardized Project Gutenberg Corpus:				
1	0.00	0.43 ± 0.47	14.08	2 ± 29
3	0.00	0.28 ± 0.43	29.06	0.4 ± 2.6
9	0.00	0.26 ± 0.44	20.27	0.5 ± 3.2
27	0.37	0.25 ± 0.48	17.21	0.6 ± 4.6
Human vs. LLM Text Corpus:				
1	0.00	1.4 ± 1.1	0.03	11 ± 262
3	0.00	1.3 ± 4.6	0.13	7 ± 220
9	0.00	4 ± 36	0.33	1.6 ± 7.7
27	4.20	1 ± 12	4.99	2 ± 37

## Data Availability

The code and instructions to reproduce the experiments are available at https://github.com/pawel-wieczynski/long_range_dependencies (accessed on 6 June 2025). More figures are available at https://github.com/pawel-wieczynski/long_range_dependencies/tree/main/figures (accessed on 6 June 2025).

## Abbreviations

The following abbreviations are used in this manuscript:

CC	cosine correlation
HLLMTC	Human vs. LLM Text Corpus
LLM	large language model
LRD	long-range dependence
SPGC	Standardized Project Gutenberg Corpus
SSLR	sum of squared logarithmic residuals
SRD	short-range dependence
